# Water savings and urban storm water management: Evaluation of the potentiality of rainwater harvesting systems from the building to the city scale

**DOI:** 10.1371/journal.pone.0278107

**Published:** 2022-11-23

**Authors:** Matteo Carollo, Ilaria Butera, Roberto Revelli

**Affiliations:** 1 Department of Environment, Land and Infrastructure Engineering, Politecnico di Torino, Turin, Italy; 2 FULL—Future Urban Legacy Lab, Interdepartmental Center, Politecnico di Torino, Turin, Italy; Soil and Water Resources Institute ELGO-DIMITRA, GREECE

## Abstract

The main potential benefits of rainwater harvesting, namely water saving and storm water management, are easily evaluable at a building scale when well-known behavioral models are used. However, the evaluation is often more complex at an urban scale, due to a lack of building characteristics and demographic data. In the present paper, we propose a method, which is based on the representative building concept that can be used to quantify the potential benefits of rainwater harvesting at different scales, that is, from the building scale to the district and city scales. Particular attention has been paid to the sizing of the system so that it can be used for different rainwater collection purposes. The method has been applied to the city of Turin (Italy) considering different scenarios: 1) domestic use (e.g., toilet flushing and the washing machine), where buildings are independent of each other, and 2) two public uses (the irrigation of public green areas and street washing), for which we have hypothesized that the rainwater collection takes place at a district scale. The non-potable water saving for domestic use varies across the city from 29% to 62%, according to the characteristics of the buildings, while the reduction of the flow peak conveyed to the sewerage system, during extreme storms, is quite constant (in the 57–67% range). Irrigation and street washing require a lower amount of water, thus about 80% of water can be saved, but the retention efficiency is low, and a slight reduction in the flow peaks can be expected. The aim of the methodology presented in this work is to provide a suitable decision-making tool for policy makers and urban planners to evaluate the capability and efficiency of rainwater harvesting systems for buildings, districts, and cities.

## 1. Introduction

There is no doubt that the ongoing population growth and the better socio-economic conditions, besides offering evident advantages, have also produced negative consequences on natural resources, including water [1, 2]. The increase in emissions has impacted the water cycle and the ongoing fast climate changes will likely decrease the availability of water in several countries [2]. Moreover, the increase in impervious surfaces, due to urbanization, with the consequent reduction in aquifer recharging, has modified both the surface runoff and flows directed to sewerage systems during rainfall events, thus raising the risk of urban floods [3].

An imperative need for a sustainable water management also emerges from the Sustainable Development Goals (SDGs) proposed by the United Nations, which should be reached before 2030: among others, Clean Water and Sanitation (SDG n. 6), Sustainable Cities and Communities (SDG n. 11) as well as Responsible Consumption and Production (SDG n. 12) are all related to the availability of water for everyone, a correct urban flood management, and a sustainable use of water [4].

About 70% of the water collected from rivers, lakes, and groundwater systems around the world is used for husbandry, irrigation, and food production, 20% is used to support industrial needs and the remaining 10% is used for domestic activities [2]. In the last few decades, a great deal of effort has been devoted to the implementation of Rainwater Harvesting (RWH) systems as a source of water in both urban and rural contexts, as these systems can significantly contribute to decreasing the environmental footprint [5, 6]. Storing rainwater can produce several benefits, depending on the specific use of the water: e.g., a reduction of water and energy consumption, an improvement of air quality, a mitigation of the Urban Heat Island (UHI) effect, water cycle restoring and urban flood mitigation. A list of the possible uses of rainwater is given in Table 1.

**Table 1:** Common uses of rainwater collected in an RWH system.

Main goal	Possible use	References
reduction of water consumption	domestic use (toilet flushing, washing machines)	[5, 7–12]
	irrigation of green areas	[13, 14]
improvement of the air quality	spraying water to reduce road dust	[15, 16]
reduction of energy consumption	heat pump for heating or cooling domestic water	[17, 18]
	electricity production (micro hydropower in gutters)	[19]
	heat absorption through water evaporation	[20]
mitigation of the Urban Heat Island effect	heat absorption and increase in humidity through water evaporation	[21–23]
urban flood mitigation	storm water retention	[24–26]
water cycle restoration	groundwater recharging	[27, 28]

With an effective RWH system, more than 30% of the domestic use could be satisfied by rainwater at the building scale: e.g., toilet flushing, the washing of clothes, the cleaning of floors, car washing, etc. [7–12] as could the watering of domestic green spaces [13, 14]. Interesting uses of rainwater have already been proposed for public services, such as road washing and the mitigation of road dust diffusion [15, 16], the irrigation of green public areas, the flushing of public toilets and for firefighting purposes. In these cases, an RWH system should be considered at the district scale. Rainwater has also been considered for domestic cooling systems [17, 18] and for electricity production, through the installation of micro-turbines in gutters [19]. The mitigation of the UHI effect and the related reduction of the energy demand for air-conditioning can be reached by spraying rainwater to absorb heat from the air (e.g. ornamental fountains) or over building and urban surfaces [20–23].

RWH also has positive impacts on hydraulic infrastructures. RWH systems retain water and reduce runoff, and thus lower the frequency of failures of drainage systems as well as the frequency of urban floods [24–26]. Finally, a partial restoration of the urban water cycle can be obtained by coupling RWH with such Nature-Based Solution (NBS) as Sustainable Urban Drainage System (SUDS) techniques, which allow rainwater to infiltrate into the ground, groundwater to be recharged, and the urban runoff to be reduced [27–29]).

The impact of an RWH system is mainly related to two aspects: the availability of rainwater and the water demand. The former depends on the climatic conditions (see Asadieh and Krakauer (2016) [30]), while the latter varies according to the uses of the rainwater: for example, toilet flushing and street cleaning clearly require different amounts of water and have distinctive temporal patterns. For these reasons, a detailed understanding of the possible uses of collected water is necessary to evaluate the appropriateness of an RWH system.

The behavior of RWH systems, at the building scale, has been studied extensively in the last few years (see, for example, Campisano et al. (2017) [5] and references therein). On the other hand, there is still a lack of knowledge about the impacts of RWH systems at larger scales. For instance, the reliability of RWH system at regional and country scales has usually been estimated through a per year balance between the total rainfall and the annual water consumption [31–33]. More accurate simulations of RWH performances have been conducted at urban scale, but these have been limited to multi-unit blocks or multi-family buildings [34, 35] or to a neighborhood [36–38]. Among the studies that have considered cities as a whole, mention can be made to that of Ghisi et al. (2006) [39], who used a rainfall-water consumption balance to show that the potential water saving that could be achieved in 62 Brazilian cities ranged from 34% to 92% (average 69%). Belmeziti et al. (2013) [40] proposed a method that takes into account the water usages and storage capacities of different buildings, and they showed that the results obtained in a district in Paris (France) were different from those obtained when considering the balance between the total rainfall and water consumption of the municipality. Bocanegra-Martínez et al. (2014) [41] presented an optimization model to design an RWH system based on cost minimization, and they applied this model to the urban area of Morelia (Mexico). Lúcio et al. (2020) [9] suggested a scale-adaptive methodology to evaluate the potential of rainwater harvesting at the urban scale, taking into consideration the characteristics and consumption patterns of the buildings. Islam et al. (2021) [42] analyzed the effect of climate change on the performances of an RWH system that provides water to the community of Paikgacha (Bangladesh).

In the present paper, we propose a method that can be used to quantify the efficiency of RWH systems at different scales: from the building scale to the district and city scales, using databases that contain the characteristics of buildings and demographic data together with land use data. The method has been applied to the urban area of Turin (Italy) considering different scenarios: domestic (e.g., toilet flushing and the washing machine) as well as public uses (e.g., the irrigation of public green areas and street washing). The potential reduction of the flow peak conveyed to a sewerage system during extreme storms has also been analyzed for the domestic use case. The aim of the methodology is to provide a suitable decision-making tool for policy makers, practitioners, and urban planners to evaluate the capability and efficiency of RWH systems for buildings, districts, and cities.

## 2. Materials and methods

**Fig 1:**
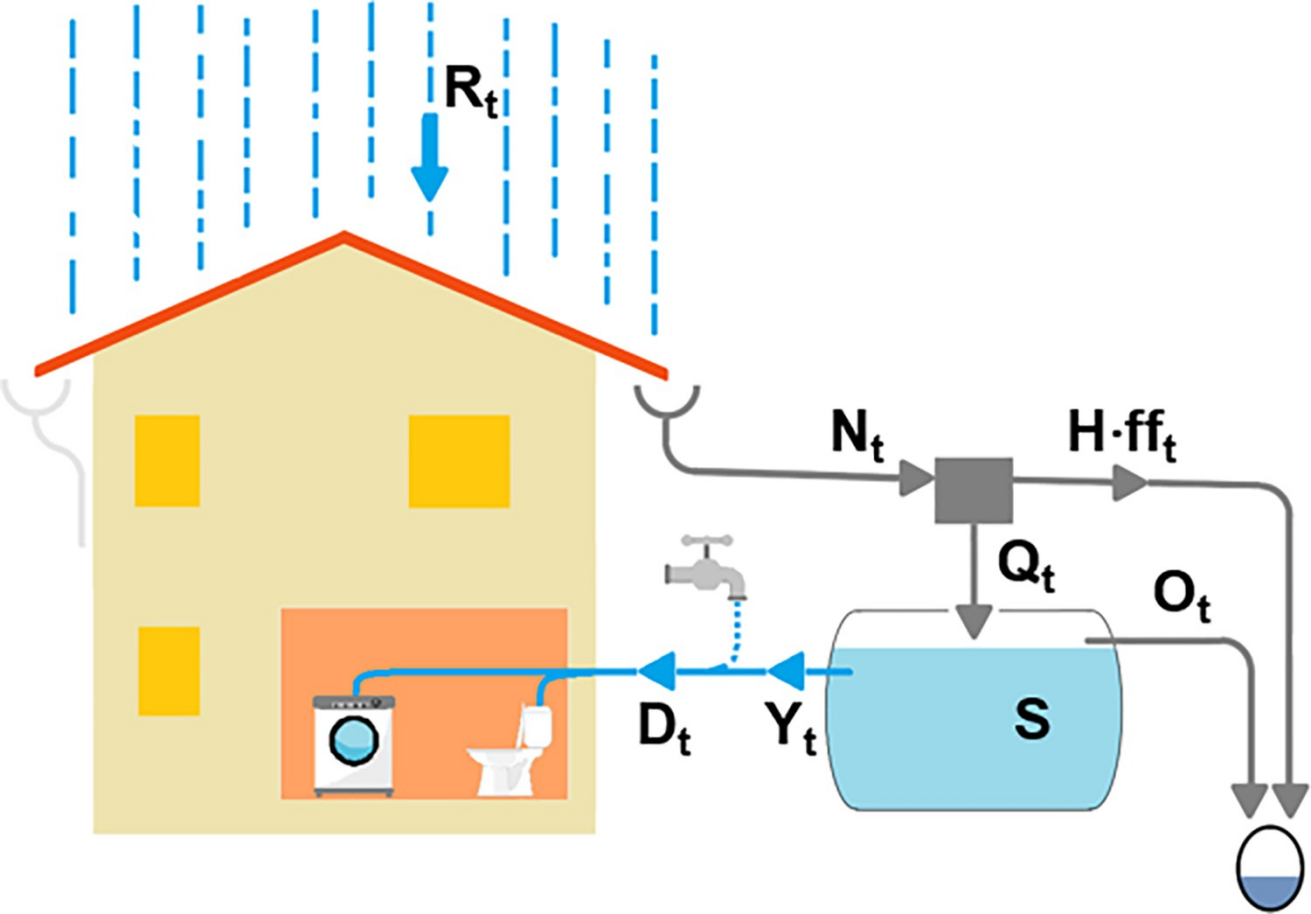
Scheme of a domestic rainwater harvesting system.

**Fig 2:**
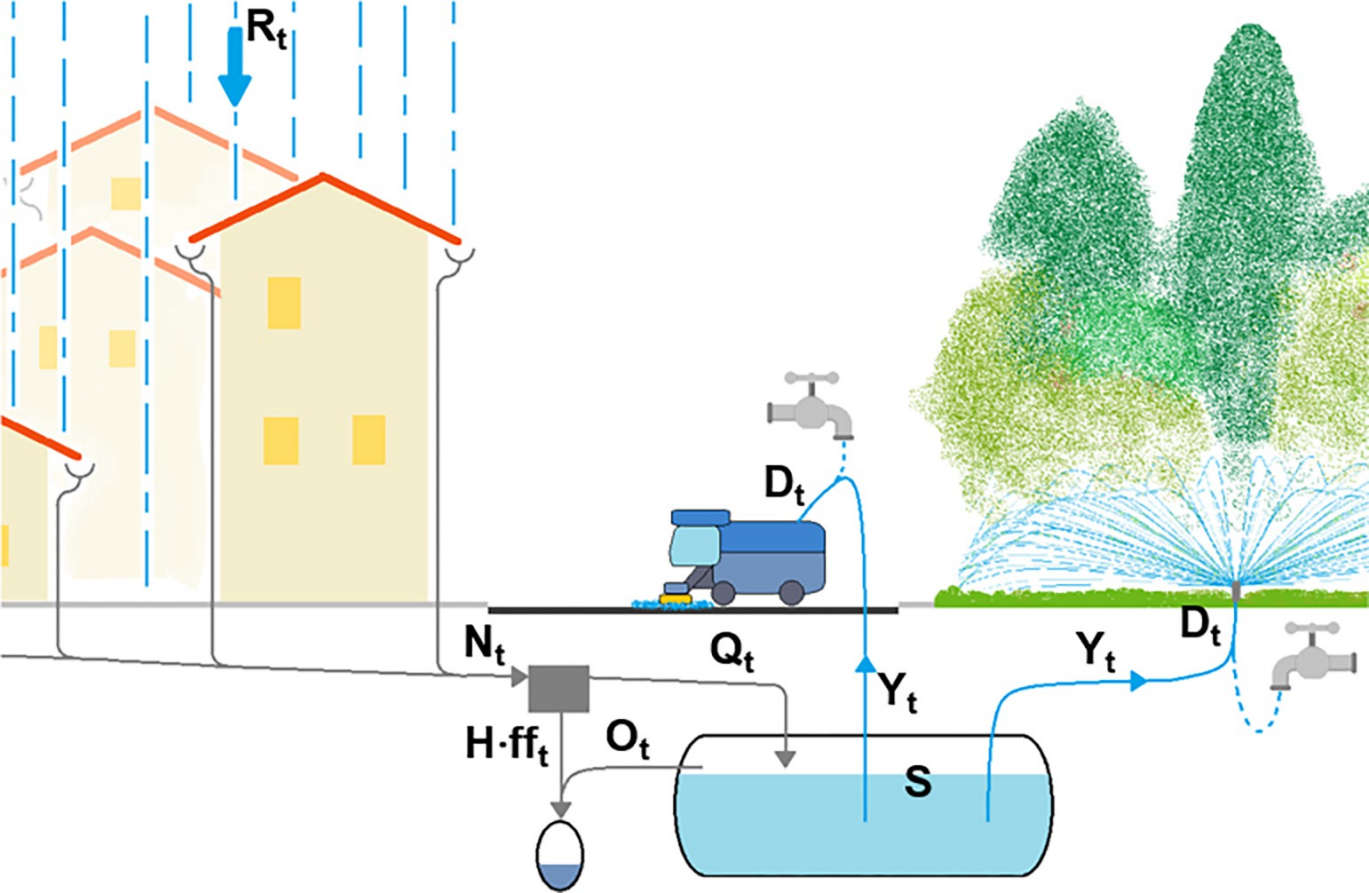
Scheme of a rainwater harvesting system for public use: Green area irrigation and street washing.

In RWH systems (Figs 1 and 2), rainfall collected by catchment surface *H*, is conveyed, through gutters, to storage tank *S*, from which a water supply system distributes rainwater to users. The tank would be useless if the water demand pattern were equal to the rainfall one, but this situation is obviously very uncommon. Moreover, the quality of the rainwater should be carefully controlled, especially for domestic use, and a device that diverts the *first flush* often has to be installed [5].

Since urban development produces a huge variety of buildings, a detailed analysis of the performance of each building is not feasible. Therefore, a method that can be used to schematize the heterogeneity of buildings should be introduced, and a group of *representative buildings* should be identified (see Lúcio et al. (2020) [9]). The performances of representative buildings can then be analyzed and extended to all the buildings they represent. In this way, it is possible to estimate the performances of an RWH system at the district or city level.

For domestic uses, a scenario is imagined in which all the buildings of the district/city are equipped with an appropriately sized RWH system. For other uses, such as the irrigation of public spaces and street washing, the analysis concentrates on the district scale, and it is assumed that a few tanks (at least one) are present in each district.

The method used to analyze the performance of an RWH system and to size it is explained in the following subsections, then the building classification approach is shown, and, finally, the method used to quantify the performances at the district and city scales is presented.

### 2.1 The RWH system: Sizing and efficiency index

Different approaches, ranging from deterministic to statistical ones, have been proposed to size and evaluate the efficiency of an RWH system (e.g., [7–9, 43–45]). Among the different methods, the so-called *behavioral models*, which are based on rain collected data and water demand patterns, make use of finite difference schemes to simulate the water balance of a tank. In this paper, we use the behavioral model proposed by Lúcio et al. (2020) [9]: unlike the widely used *Yield After Spillage (YAS)* or *Yield Before Spillage (YBS)* methods (see Fewkes and Butler (2000) [7]), this model considers that a fraction of the daily inflow is conveyed to the tank before the distribution of the daily water supply.

The core of the model (see Figs 1 and 2) is the following water balance equation for the tank:

$$V_{t}=Q_{t}+V_{t-1}-Y_{t}-O_{t}$$ (1)

where *V*_*t*_ is the volume of water contained in the tank after the inflows and outflows that occur in interval *t*, *Q*_*t*_ is the rainwater volume that enters the tank, *Y*_*t*_ is the rainwater volume supplied to the users and *O*_*t*_ is the overflow volume. The subscript *t* refers to a generic time step, 1<*t*<*T*, where *T* is the total simulation period. The duration of the time step depends on the aim of the simulation: a daily time step is usually considered for a water saving analysis [5, 8, 46], while a one-minute time step is used for the analysis of the reduction of runoff peaks [24]. The model is solved through a finite-difference scheme.

As far as the inflow to the tank is concerned, not all the rainwater that falls onto a roof surface can be used, because a certain amount of water is lost through evaporation or is diverted through first flush diversion due to its low quality. Hence, the inflow is computed as:

$$Q_{t}=(\varphi ∙R_{t}-{ff}_{t})∙H=N_{t}-{H∙ff}_{t}$$ (2)

where *R*_*t*_ is the rainfall depth, *φ* is the runoff coefficient and *ff*_*t*_ is the first flush diversion. In this way, *N*_*t*_ = *φ*∙*R*_*t*_∙*H* is the net rain volume. Typical values of *φ* and of the first flush are 0.8 [46, 47] and 1 mm per rainy day [48, 49], respectively. In the case of a runoff reduction analysis, where the time step is short, *Q*_*t*_ is zero in the earlier time intervals, as long as the sum of the consecutive *ff*_*t*_ is lower than 1 mm.

The terms *Y*_*t*_ and *V*_*t*_ in Eq (1) are computed with the following algorithm [9]

$$Y_{t}=min\left\{ \begin{array}{c} D_{t}\\ V_{t-1}+\alpha ∙Q_{t}\\ S \end{array}\right.\;and\;V_{t}=min\left\{ \begin{array}{c} V_{t-1}+Q_{t}-Y_{t}\\ S-Y_{t}+(1-\alpha )∙Q_{t}\\ S \end{array}\right.$$ (3)

where *D*_*t*_ is the non-potable water demand, which depends on the use patterns (e.g. domestic, irrigation, street washing, etc.), and the coefficient *α* represents the fraction of rainwater conveyed to the tank before the daily supply (*α* varies between 0 and 1). Preliminary results, not shown here for the sake of brevity, indicated that, in our case study, the *α* value has little impact on the RWH performance indexes. For this reason, we chose *α* = 0.5, whereby half of the net rainwater volume collected on the roof is conveyed to the tank before the daily supply.

Two quantities are computed for each time step to evaluate the retention efficiency of RWH systems and the reduction of runoff peaks:

$$sw_{t}=Q_{t}-O_{t}$$ (4)

$$F_{t}=O_{t}+{H∙ff}_{t}$$ (5)

where *sw*_*t*_ is the volume of the retained rainwater, and *F*_*t*_ is the volume of the rainwater discharged into the sewerage system.

Two additional quantities are required for the following analysis: the annual rainwater volume collected on roof *Q*, which is computed considering only the runoff coefficient and neglecting the first flush, and the annual non-potable water demand *D*.

#### 2.1.1 Size of the tank

In theory, an optimal tank should be a tank for which the water saving efficiency *E*_*WS*_ is maximum. *E*_*WS*_ represents the ratio between the total rainwater volume supplied to the user to the total demand for specific use

$$E_{WS}=\frac{\sum_{t=1}^{T}Y_{t}}{\sum_{t=1}^{T}D_{t}}∙100$$ (6)

i.e., the higher the efficiency is, the larger the volume of saved potable water. Water saving efficiency is evaluated for a period long enough to represent the hydrologic regime and assuming an empty tank at the beginning of the period [8, 46, 50]. Different tank capacities are considered, and the minimum capacity should respect the *S*/*Q*≥0.01 constraint, as a daily time step is used [7].

The water saving efficiency, *E*_*WS*_, grows according to the tank capacity: it first grows remarkably fast as function of the tank capacity, and then the increase progressively slows down. The maximum water saving efficiency is obtained for very large tanks, which means both a difficult installation and perhaps even economic unfeasibility.

In practical situations, a water saving efficiency that is below the maximum achievable often has to be accepted, and a rule to choose the most appropriate tank size is therefore needed. In the case of the domestic use of rainwater, as a first step, we adopted the Italian Standard method [46, 51]: tank capacity *S* is 10% of the minimum between the annual inflow *Q* and the annual demand *D*. Subsequently, we checked that the water saving efficiency obtained with this criterion was satisfactory, that is, at least 80% of the maximum achievable [30, 51]: if the test had failed, a larger tank would have been considered until 80% of the maximum *E*_*WS*_ was reached.

We found no rules in the literature for the other considered rainwater uses (irrigation and street washing), and we therefore selected the tank size for which the water saving efficiency is 80% of the maximum value.

Once the tank size had been defined according to the above-described rules, it was considered appropriate to evaluate the retention efficiency of the RWH system as [9, 24]:

$$E_{R}=\frac{{\sum_{t=1}^{T}sw}_{t}}{H\sum_{t=1}^{T}R_{t}}∙100$$ (7)

However, it was not possible to reach a retention efficiency of 100%, due to the presence of first flush diversion and to the loss of water through evaporation or retained by the roof. In fact, the retention efficiency of a tank increases as the volume of water stored in the tank increases. Moreover, it is null when the tank does not store water, for instance when the rain inflow is equal to the first flush.

#### 2.1.2 Reduction of the runoff discharge

A comparison between the maximum flow discharged during intense storms, with and without an RWH system, should be made to quantify the reduction of the runoff discharge to the sewerage system. According to Campisano and Modica (2015) [24], the efficiency of the peak reduction per building and for a storm event is given by:

$$E_{PR}=\left(1-\frac{F(t_{r})}{N(t_{p})}\right)∙100$$ (8)

where *F(t*_*r*_*)* is the maximum rainwater discharge released by a building equipped with an RWH system and *N(t*_*p*_*)* is the maximum discharge which, in the absence of an RWH system, would flow directly to the sewerage system.

The *t*_*r*_ and *t*_*p*_ times can be different, due to the presence of a tank, and, consequently, the efficiency of the peak reduction is a function of the tank size, the rain intensity, and the level of water in the tank at the beginning of a storm. A short time step, i.e. one minute [24], should be considered to analyze the behavior of the tank during a storm event, and the initial water level in the tank is provided by the previous simulation that adopts a daily time step. The first flush diversion should be considered carefully: we have assumed that the first flush is conveyed to the sewer at the same time than it occurs, hence, an RWH system is not able to reduce the runoff at the beginning of a storm.

### 2.2 Choice of representative buildings

It is not feasible to consider buildings one by one to analyze the performance of an RWH at the district or city scale, and it is more convenient to work with classes of buildings. Each class is identified by a *representative building*, and an appropriate number of representative buildings should be chosen. Lúcio et al. (2020) [9], for instance, suggested a number of representative buildings that represented at least 40–50% of the total building stock. In our work, we focused on classes with a higher frequency than 10%.

Three parameters are considered to identify a representative building: the roof area (i.e., harvesting area), the inhabitants (related to the water demand) and the number of buildings that it represents in the specific class. Unfortunately, data about building roof areas and the inhabitants of buildings are often lacking in ready-to-use statistical classifications, and metadata often have to be used to identify representative buildings: e.g., the number of apartments, the number of floors, the district population, the number of families and the average area of the apartments.

### 2.3 Performance at the district and city scales

The evaluation of the water saving and runoff reduction of a building can now be extended to the district and city scales. If the water saving efficiency is computed for each representative building, the water saving efficiency, $E_{WS}^{*},$ can be obtained for an ensemble of buildings (i.e., a district or a city) from the ratio between the volume of rainwater supplied to the users to the volume of requested rainwater in the examined reference period

$$E_{WS}^{*}=\frac{\sum_{k=1}^{K}n_{k}∙\sum_{t=1}^{T}Y_{kt}}{\sum_{k=1}^{K}n_{k}∙\sum_{t=1}^{T}D_{kt}}∙100=\frac{\sum_{k=1}^{K}{E_{WS}}_{k}∙D_{k}∙n_{k}}{\sum_{k=1}^{K}D_{k}∙n_{k}}∙100$$ (9)

where *K* is the number of building classes at the considered scale, *D*_*k*_ is the volume of rainwater requested in the reference period by the representative building for the *k* building class, *n*_*k*_ is the number of buildings that belong to the *k* class, and ${E_{WS}}_{k}$ is the corresponding water saving efficiency. The efficiency of RWH systems at the considered scale depends on the water saving efficiency, the rainwater demand and the numerousness of each representative building.

The same method is applied to evaluate the reduction of the peaks of the runoff discharge introduced into the sewerage system. The volumes discharged by all the buildings in the district/city are summed at each time step, in both the presence of RWH and in its absence. In this way, the efficiency of the peak reduction can be quantified through

$$E_{PR}^{*}=\left(1-\frac{F^{*}(t_{r})}{N^{*}(t_{p})}\right)∙100$$ (10)

where F* is the highest flow rate discharged by all the buildings supplied by RWH systems, N* is the highest rainwater flow rate in the absence of RWH systems, and *t*_*r*_ and *t*_*p*_ are their time peaks, respectively. The $E_{PR}^{*}$ index is computed for each storm event and the mean value is then computed.

## 3. Non-potable water demand

Quantification of the non-potable water demand is a key issue in assessing both the economic convenience and the hydraulic efficiency of an RWH system. In this study, three rainwater use scenarios have been considered: (1) domestic use (mainly toilet flushing and washing clothes), (2) the irrigation of public green areas and (3) street washing.

The domestic use is evaluated as the product between the non-potable water demand per capita and the number of people, *P*, living in each building. A daily demand pattern is used to size the rainwater storage tank, while a sub-daily demand pattern is considered during the analysis of the runoff peak reduction.

The water demand for irrigation and street washing is based on the extension of the irrigated and street surfaces, as well as the specific water demand (l/m^2^ or mm). Several methods (e.g., Penman-Montheit, Hargreaves) have been developed to estimate the specific water demand of crops as a function of the soil moisture, evapotranspiration, and rainfall [52, 53], and a daily time step can be used to describe the variation of all these quantities over the year. However, for the purposes of this paper, we have evaluated the specific irrigation demand as the difference between the daily evapotranspiration and rainfall, and a negligible retention capacity of the soil has been considered. The specific water needs for street washing depend on the municipality: in this work, the washing frequency and water demand have been based on information made available by the local waste management company.

## 4. Case study

The case study focuses on Turin (northern Italy—45°04’N 7°42’E), an Italian city of about 870000 inhabitants located at a mean altitude of 240 m a.s.l. The climate in Turin (*Cfa*, Humid subtropical climate—according to the Kӧppen-Geiger classification) is temperate, without a dry season, and it is characterized by a hot summer (the average temperature in the hottest month is more than 22°C), while winter is the driest season. Precipitations are concentrated in two periods, the first ranges from April to June and the second is in November, with an annual average rainfall depth of about 850 mm.

**Fig 3:**
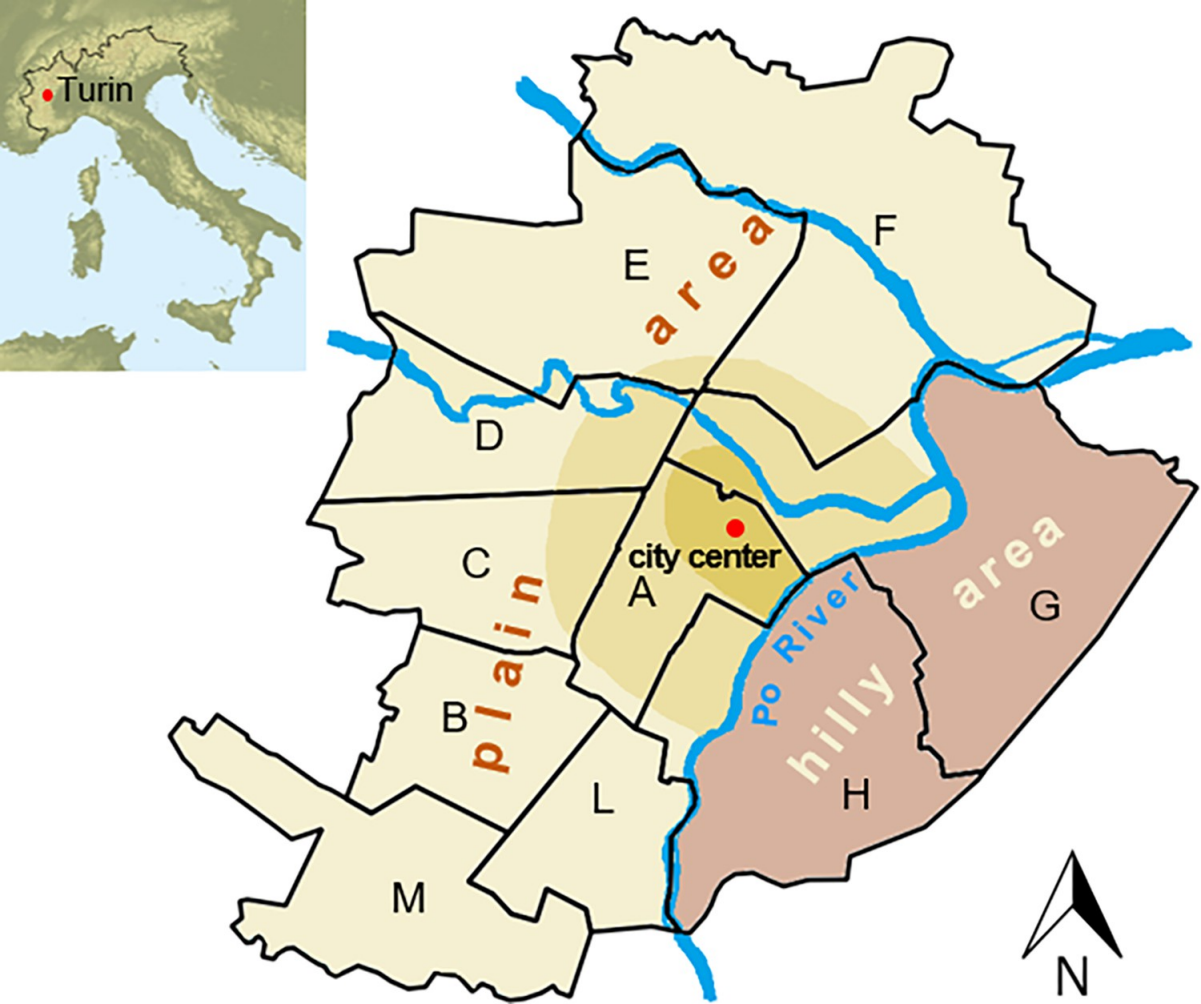
The city of Turin and its administrative districts.

The city of Turin (Fig 3) is characterized by a part on the plain, on the west, and a hilly part (max 600 m a.s.l.), on the east (eastern parts of districts G and H), and these two parts are divided by the River Po, which flows from South to North. The city occupies a land area of about 130 km^2^, two-thirds of which are urbanized, while the last third is composed, by two-thirds, of private and public green areas and the remaining part of woods, which are mostly widespread in the hilly part of the city [54, 55]. The part of the city on the plain appears to be almost completely urbanized and is organized in a regular network of streets and avenues, while the hilly part is characterized by a quite steep slope and some small valleys, with many woods and meadows.

In this paper, we have considered the administrative subdivision of the city [56], which considers the presence of ten districts (Fig 3), to take advantage of existing databases. The northern part of district A is the city center, that is, the ancient part of the city (in a very recognizable Roman style) together with the most western part of district G: this area is characterized by low-rise buildings (max. six floors) with a large floor extension and few green areas. In a more external belt, which comprehends the southern part of district A, the western part of H, the most eastern part of C and D, and the western part of G, urbanization is more recent (Industrial Revolution, late 1800s - early 1900s), while the remaining districts in the plain area of the city were mainly built after World War II, especially during the Economic Boom (from the 1950s to 1970s): in this case, the distribution of buildings is more heterogeneous than in the old part of the city, with both low-rise buildings (1 floor) and high-rise buildings (up to 15 floors). Urbanization is quite scarce on the hilly side of the city (G and H on the east): several ancient and more recent country houses are present in this hilly environment, as well as detached houses and blocks of flats, often with private gardens.

Table 2 shows data collected for the different districts which are used in the next sections. The public green spaces include several elements (e.g., parks, gardens, flowerbeds, avenues with trees and woods) that are partially irrigated by the municipality (in most cases, using drinking water). In this work, we have focused on the irrigation of the park area with RWH systems. The extension of the irrigated park areas is a datum that is only available at the city scale: for this reason, the ratio between the irrigated park area and the total park area in each district has been assumed equal to the ratio computed for the whole city. The irrigated area of the city is about 1 km^2^ [57] and the total parks area is about 6.9 km^2^ [58].

**Table 2:** Main features of the municipal districts.

	municipal districts										
	A	B	C	D	E	F	G	H	L	M	Total
quarters	Centro Crocetta	Santa Rita Mirafiori Nord	Borgo San Paolo Cenisia Cit Turin Pozzo Strada	San Donato Parella Campidoglio	Borgo Vittoria Madonna di Campagna Lanzo Lucento Le Vallette	Regio Parco Barca Bertolla Barriera di Milano Rebaudengo Falchera Villaretto	Aurora Vanchiglia Sassi Madonna del Pilone	Borgo Po San Salvario Cavoretto	Nizza Millefonti Lingotto Filadelfia	Mirafiori Sud	
land area (km^2^)	6.89	7.31	8.60	9.09	15.57	25.52	22.53	16.53	6.49	11.86	**130.39**
population (inhabitants)	75966	100574	127187	95238	121608	102188	83381	55109	73095	38021	**872367**
mean family size	1.9	2.1	2.0	2.1	2.2	2.2	2.1	2.0	2.1	2.3	**2.1** ^a^
roof area *H* (km^2^)	1.58	0.94	1.43	1.26	1.30	1.64	1.49	1.30	0.78	0.50	**12.2**
irrigated area *Ir* (10^3^ m^2^)	18	45	19	145	61	142	179	272	63	96	**1040**
*H/Ir* (-)	88	21	75	9	21	12	8	5	12	5	**11.7** ^b^
street area *St* (km^2^)	1.34	1.16	1.46	1.23	1.78	2.21	1.59	1.14	0.76	1.08	**13.8**
*H/St* (-)	1.2	0.8	1	1	0.7	0.7	0.9	1.1	1	0.5	**0.89** ^b^

a) Weighted arithmetic mean with the population as a weight

b) Ratio between the total areas

There are few parks in the oldest parts of the city (district A), while there are frequently very large parks in the most recent districts (e.g., the large “Parco della Pellerina” in district D, near the Dora River). Moreover, the parks in the hilly districts (G and H) are extremely large.

As far as the RWH for street washing is concerned, the areas used for vehicular traffic (streets, roads, squares, etc.) in each district were considered. Their extension was obtained from land use digital maps [55] and the overall area amounts to 13.8 km^2^ for the whole city.

In this paper, residential buildings have been considered, in terms of collected rainwater for domestic use, park irrigation and street washing. Residential buildings play an important role in the Turin urban context and they cover up to 50% of the land area occupied by all the buildings.

The representative buildings were selected using land use digital maps [55] as well as population statistics and a building census [59]: i.e., the population and number of families, classification of the buildings according to the number of floors, and the number and average area of the apartments.

After several tests, we chose (Table 3) from 3 to 6 representative buildings per district: each representative building was selected in such a way that it was representative of at least 10% of the buildings in the district. Table 3 also shows the inhabitants *P* and the roof area *H* of each representative building, as well as the percentage of the buildings in each district that correspond to each representative building type. It was also checked that both the total population and the total roof areas, that is, according to our choice of the representative buildings matched the total roof area and population data in each district. The estimated values were computed by multiplying the population and the roof area of each representative building by the number of the buildings it represented. In this way, the water demand and collected rainwater volume obtained from our model matched the available data for each district.

**Table 3:** Total number of buildings in each district; frequency (%—plain), population *P* (inhabitants—bold) and roof area *H* (m^2^—italics) of the representative buildings in each district.

	municipal district									
	A	B	C	D	E	F	G	H	L	M
Number of buildings	3110	2768	5144	4203	4452	4668	4416	4311	1747	1339
	13%	12%	22%	11%	12%	15%	13%	25%	13%	19%
	**11**	**6**	**12**	**6**	**2**	**2**	**2**	**2**	**12**	**2**
	*240*	*200*	*200*	*200*	*100*	*100*	*100*	*120*	*190*	*10*0
	34%	18%	38%	20%	11%	13%	15%	18%	25%	15%
	**22**	**12**	**24**	**12**	**4**	**4**	**4**	**4**	**25**	**5**
	*460*	*200*	*300*	*200*	*100*	*100*	*100*	*120*	*385*	*100*
	53%	23%	40%	35%	12%	14%	14%	16%	62%	13%
	**29**	**25**	**32**	**25**	**7**	**7**	**6**	**6**	**55**	**7**
	*600*	*200*	*300*	*200*	*200*	*200*	*200*	*240*	*520*	*200*
		47%		34%	18	18%	15%	12%		14%
		**59**		**32**	**13**	**13**	**13**	**12**		**14**
		*500*		*500*	*200*	*200*	*200*	*240*		*200*
					19%	16%	17%	12%		13%
					**26**	**27**	**25**	**24**		**27**
					*200*	*400*	*400*	*475*		*400*
					28%	24%	26%	17%		26%
					**65**	**56**	**42**	**38**		**79**
					*600*	*800*	*700*	*750*		*900*

### 4.1 Domestic use of rainwater

In this scenario, all the buildings in Turin have been considered to be equipped with a tank, and domestic use of the rainwater has been assumed. The non-potable water demand per capita for domestic use was considered equal to 50 liters/(person·day), and constant during the year [46]. On the basis of these hypotheses, the amount of non-potable water needed in Turin was calculated to be about 16·10^6^ m^3^ per year.

A thirty-year-long rainfall pattern was considered for the simulations for the whole city, using the data collected at two rain-gauge stations managed by the regional Environmental Protection Agency [60].

#### 4.1.1 The best tank design and water savings

The tank volumes were selected after a series of simulations, as explained in Section 2.1.1. The best tanks were distributed throughout the city in the following way: 7–10 m^3^ for 16% of the buildings, 12–18 m^3^ for 35% of the buildings, 20–35 m^3^ for 21% of the buildings, and 35–63 m^3^ for 28%. The sum of the tank capacities over the whole city is about 8.65·10^5^ m^3^.

**Fig 4:**
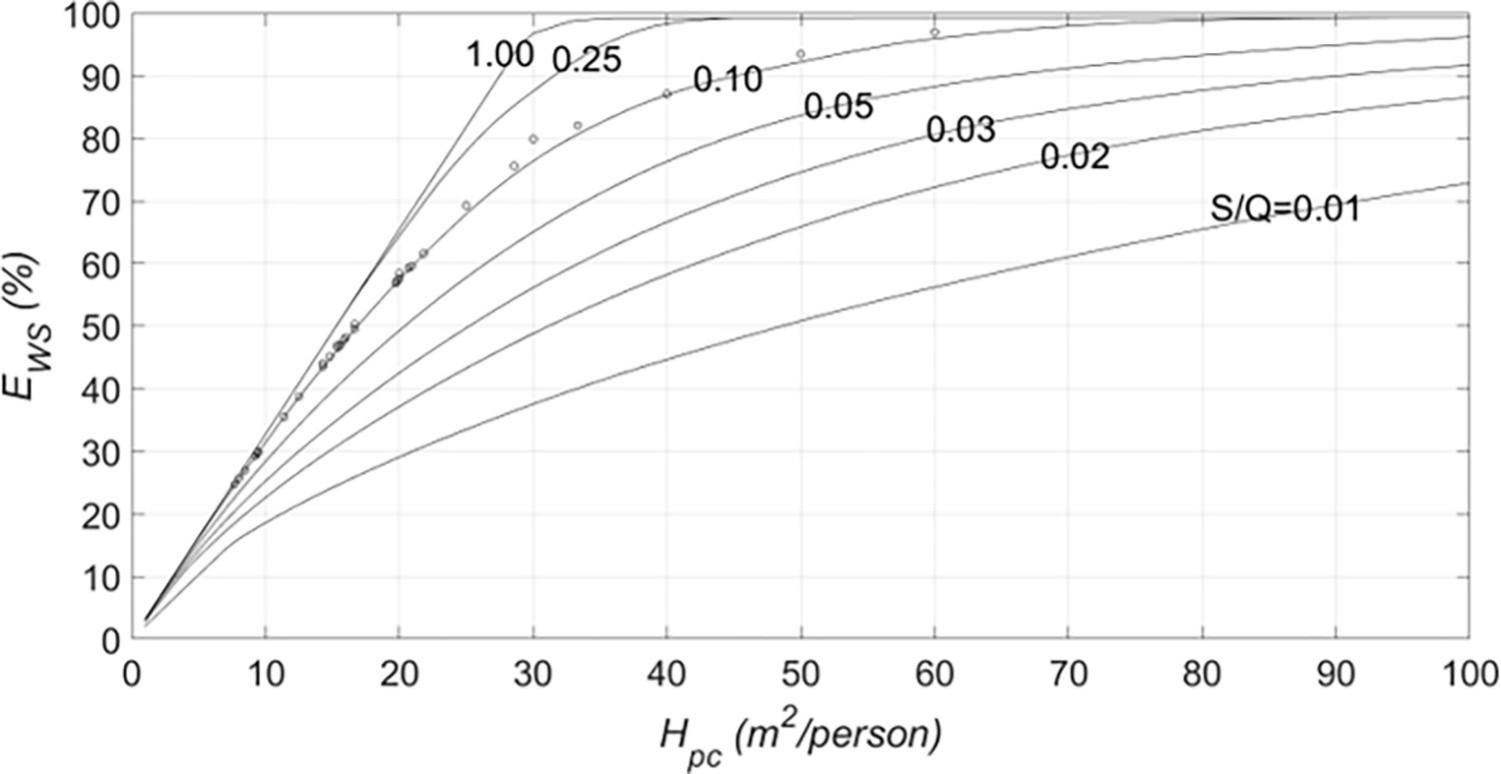
Water saving efficiency as a function of the roof area per capita for different storage fraction values. The dots represent the performances of the representative buildings.

The parameters that mainly affect the water saving efficiency of a building are the tank capacity and the harvesting area per capita, *H*_*pc*_ = *H*/*P*, which is proportional to the ratio between the rainwater availability and the water demand. The water saving efficiency is shown in Fig 4 as a function of the roof area per capita for different storage fraction values. The lines in Fig 4 were obtained by simulating Eqs (1) to (3) with Turin rain data. *E*_*WS*_ grows according to *H*_*pc*_ for a fixed *S/Q* value. The circles in Fig 4 represents the results obtained from simulations of all the representative buildings in the districts, according to the tank sizing rule described in Section 2.1.1. As can be seen, most circles are very close to the *S/Q* = 0.1 curve suggested by the Italian Standards method (Section 2.1.1). It is worth recalling that the water saving efficiency reached by each representative building is at least 80% of its maximum value. Most representative building data are characterized by values of *H*_*pc*_ in the 10–25 m^2^/person range and by water saving efficiencies of between 30% and 70%.

The curves in Fig 4 come close to each other for small and large *H*_*pc*_, which shows that the tank size is irrelevant for *E*_*ws*_ when the roof area is very large with respect to the inhabitants, because the rainwater inflow is high, or when *H*_*pc*_ is very small, i.e., there is just a slight need of storage [8, 14].

**Fig 5:**
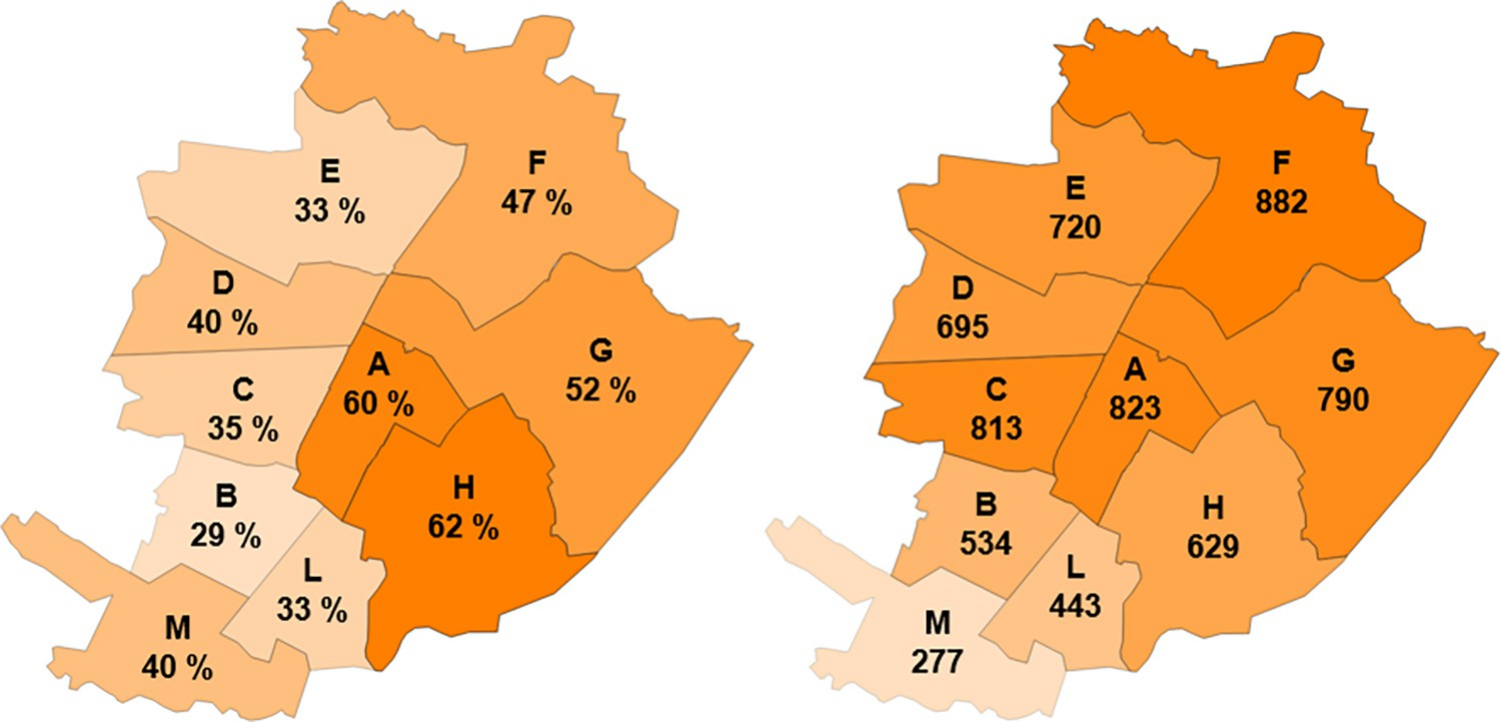
Domestic use of rainwater. Left: water saving efficiency. Right: average annual volumes of saved water (thousands of m^3^ per year).

It has been deemed interesting to map the water savings efficiency at a district level (Fig 5 - left), to denote the impact of different urbanistic systems as well as the zones where RWH is more efficient. The color scale refers to the minimum and maximum reported values (the same criterion is used in the following). The water saving efficiency varies over the 29–62% range: high values are achieved in districts with high *H*_*pc*_, such as the districts in the hilly part or in the ancient part of the city (A, F, G, H) where family houses or quite low-rise buildings (maximum 5 floors) are very common. Nevertheless, the district water saving not only depends on *H*_*pc*_, but also on the rainwater demand of each representative building (see Eq (9)): for instance, the most frequent building (one-family house) in district H has a high efficiency (97%), but a lower water need, than other representative buildings, thus the district efficiency reaches just 62%.

The water savings efficiency for the whole city is 42%, which means that almost half of the non-potable water needs of residential houses could be satisfied by rainwater harvesting. In terms of volume, the city of Turin could save about 6.6·10^6^ m^3^ of drinking water in one year, distributed over the districts according to Fig 5 (right).

**Fig 6:**
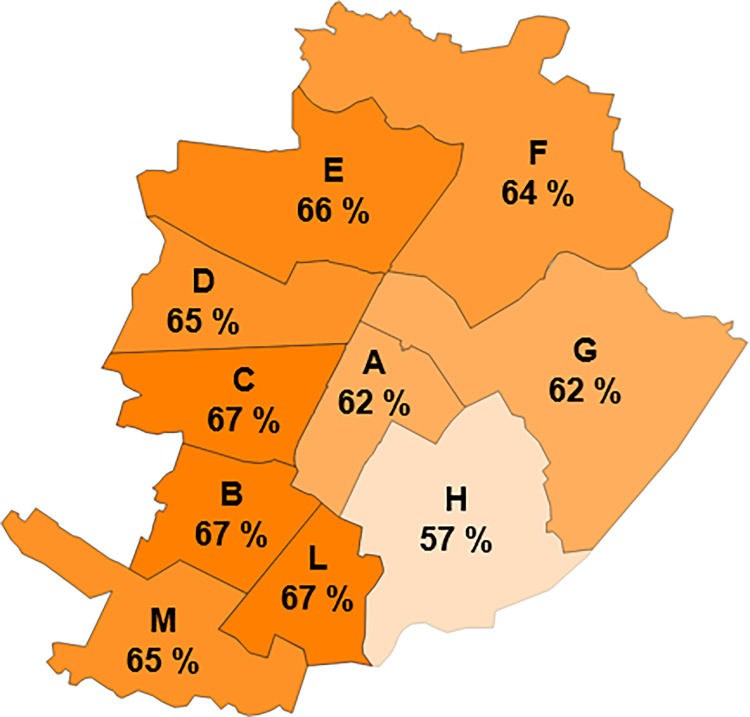
Domestic use of rainwater. The retention efficiency of each district.

It is also interesting to observe the retention efficiency (Fig 6): the quantity of collected rainwater is about 65% of the amount of rainwater that falls onto the roofs in Turin, in the reference period, and this is a volume of rainwater that is not conveyed to the sewerage system.

#### 4.1.2 Reduction of the sewage flow due to the domestic use of rainwater

The scenario presented in Section 4.1.1, with all the residential buildings collecting rainwater for domestic use, is now examined focusing on the reduction of runoff from residential buildings and conveyed to the sewage system.

A time step of one minute has been adopted for this analysis and 23 storm events have been identified from the rain-gauge station data of Turin [60]. These storms are the annual events with the highest 10-minutes intensity. On the basis of the available information on domestic water use, the sub-daily water demand pattern has been drawn up for the simulation with a time step equal to 1 minute. The selected pattern shows two peaks (at 9 a.m. and at 9 p.m.) with a peak coefficient *p*_*c*_ = 2.5 (i.e., the ratio between the peak of the flow rate and the daily mean value).

**Fig 7:**
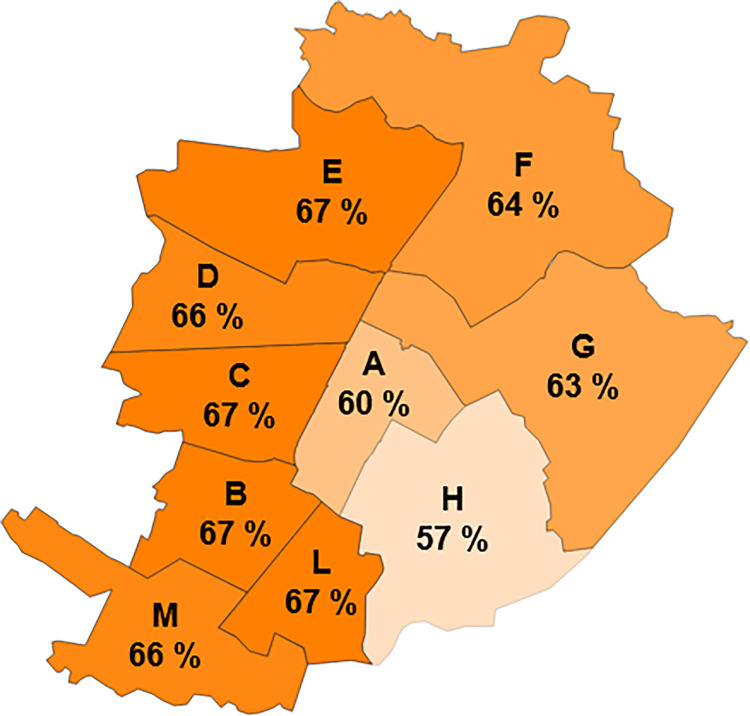
Domestic use of rainwater. The runoff peak reduction efficiency for each district. The map shows the mean value of 23 storm events.

The results of the simulations are shown in Fig 7 for each district, where the average runoff peak reduction efficiency for the 23 storm events has been mapped. The peak reduction efficiency obtained from the residential buildings is quite uniform, varying between 57% and 67%. It can be observed that the presence of the first flush diversion can affect the results to a great extent, because the rainwater collected in the first stages of the storm (when it is most probable that the rainfall peak occurs) is all conveyed to the sewage system, thus, the RWH system is likely to be inactive during the initial phase of the storm.

### 4.2 Public use of rainwater: Irrigation and street washing

The rainwater collected on the roofs of residential buildings can also be used for public purposes. We have assumed that, in the case of the irrigation of public areas (parks, flowerbeds, etc.) or street washing, rainwater is stored in just a few large tanks (at least one) placed in appropriate locations (e.g., they could be near the parks of interest for irrigation purposes) where water can easily be withdrawn and conveyed to the irrigation plant or to tank trucks for street washing. An important difference between domestic and public use is that, in the latter case, rainwater can be used far from where it is collected and it may not be necessary to involve all the buildings in the collection of rainwater to avoid the construction of an extended collection network that might not be feasible.

The harvesting area can be chosen by policymakers who should balance the extension of the rainwater catchment and the size of the tank to obtain the desired water saving efficiency; to this aim, useful diagrams are provided in the following sections.

#### 4.2.1 Irrigation of urban green spaces

The irrigation of green areas is one of the activities that can involve rainwater; the necessity of the first flush diversion should be discussed. However, the first flush diversion has been considered in this study. For simplicity, irrigated public green areas, which are usually composed of a mixed group of plants (e.g., grass, trees, bushes, flowers, etc.), are considered as meadows. The water demand for irrigation is quantified using the simple pattern of the meadow evapotranspiration proposed by Buzzacchi et al. (2008) [61] in a study on the Po River basin: i.e., 2.4 mm/day in April, 5.8 mm/day from May to July, 4.8 mm/day in August and 3.7 mm/day in September. The irrigation system, which could be a sprinkler irrigation plant, is considered to be completely efficient with negligible water losses. The water requirements for the irrigation of the whole city amount to about 720·10^3^ m^3^ per year, but are concentrated in the April to September period.

**Fig 8:**
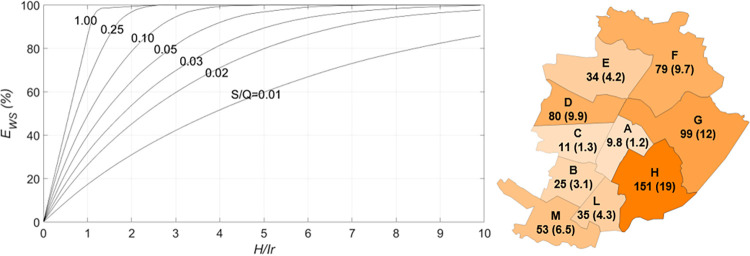
Irrigation. Left: dimensionless graph used for tank design purposes. Right: average water savings in one year (10^3^ m^3^) and the required capacity of the tanks, in brackets (10^3^ m^3^).

The diagram in Fig 8 (left) proposes a tool for the design of RWH tanks. This tool is the result of simulations of different *S*/*Q* values with reference to the thirty year-long rainwater series. The ratio between the rainwater catchment area and the area to be irrigated is shown on the horizontal axis. After several trials, we selected a water saving efficiency of 80% and a storage fraction of 0.02 to have a good water saving efficiency and reasonable tank size, so that the *H/Ir* ratio is about 5. It is worth observing that the maximum obtainable $E_{WS}^{*}$ of RWH for irrigation purposes can reach 100%.

It results, from the data in Table 2, that just a few buildings in some districts (e.g., districts A and C) need to be involved in rainwater harvesting for park irrigation purposes; in fact, *H/Ir* is larger than 5 if all the district building are considered. In other districts (e.g., H and M), the whole catchment surface *H* has to be used.

Fig 8 (right) shows the water savings per year and the required capacity of the tanks, for the selected efficiency $E_{WS}^{*}$ equal to 80%. The average water saving for the whole city is 577·10^3^ m^3^ per year and the total storage capacity required is about 71·10^3^ m^3^, that is, less than 1/10 of the storage capacity for domestic use. With these tanks, the retention efficiency of the city is about 6%. The expected low value of retention efficiency is a clear indicator of a low capacity to reduce the runoff peak.

#### 4.2.2 Street washing

Street washing in Turin is carried out by tank trucks that spray high-pressure water over the road surface. A water consumption of 0.2 l/m^2^ and a cleaning frequency of once a week (on Wednesday) have been hypothesized in this study, on the basis of the information made available by the local waste management company. The water requirements of the whole city amount to about 143·10^3^ m^3^ per year.

**Fig 9:**
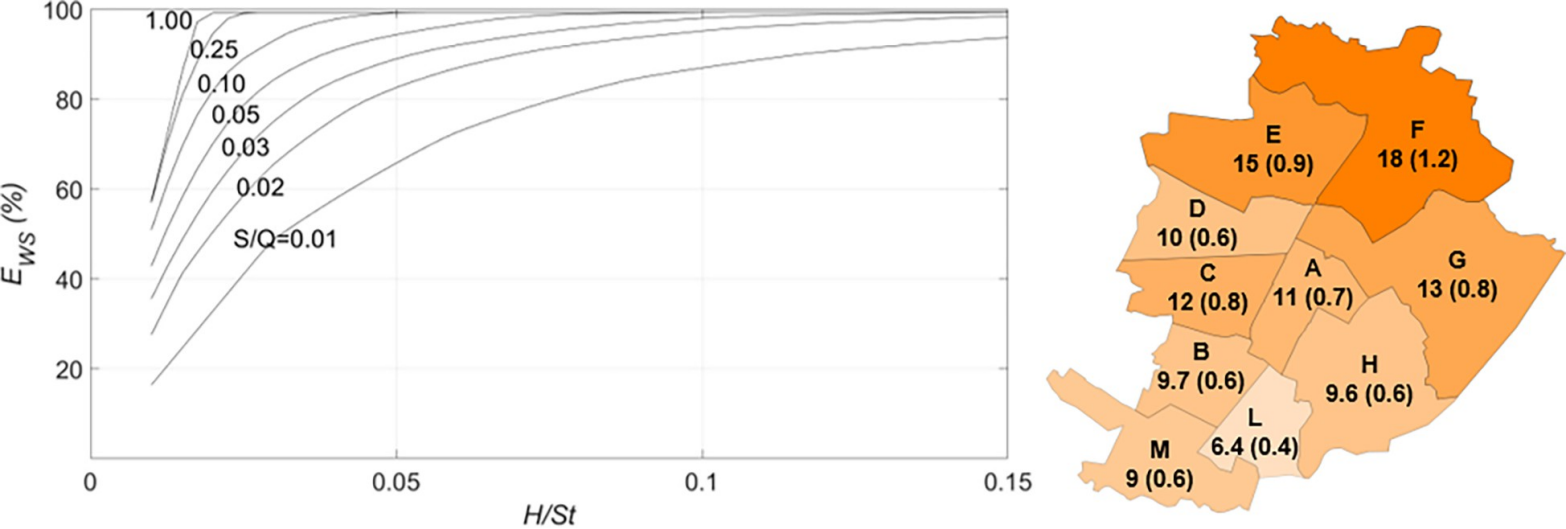
Street washing. Left: dimensionless graph used for tank design purposes. Right: average water savings in one year (10^3^ m^3^) and the required capacity of the tanks, in brackets (10^3^ m^3^).

A diagram like those in Figs 4 and 8 has been made to decide how many buildings need to be involved in the rainwater harvesting (Fig 9, left). In this case, the value of *H/St* is shown on the horizontal axis, where *St* is the washed street area. After several trials, we selected a water saving efficiency of 80% and an *S/Q* = 0.01 to obtain a good water saving efficiency and tanks that were not too large, thus the required *H/St* is 0.075. As in the case of RWH for irrigation purposes, the maximum obtainable $E_{WS}^{*}$ can reach 100%. Fig 9 (right) shows the mean volume of water saved per year and the storage capacity required for each district. The water saving at the city scale is 115·10^3^ m^3^ and the required storage capacity is 7.2·10^3^ m^3^. As expected, the retention efficiency of the whole city is very low (about 1%).

## 5. Conclusions

This paper analyzes the potential reduction of the consumption of drinking water as a result of rainwater harvesting in the city of Turin in three scenarios: 1) all the residential buildings are equipped with an appropriate tank for domestic use (toilet flushing and washing clothes), 2) the irrigation of green public spaces, and 3) street washing, using only a few public tanks and involving only a certain number of buildings. Moreover, the domestic use scenario has also been studied pertaining to storm water management, focusing on the reduction of the discharge peaks introduced into the drainage system. A simplified schematization of the city has been used, considering just a few types of representative buildings.

The non-potable water requirement for domestic use is the largest of the three considered uses and, at the city scale, rainwater can substitute just 42% of this, which corresponds to 15% of the overall domestic water demand. Different water saving efficiencies have been reached for the municipal districts, mainly as a function of the roof area per capita, with higher efficiencies wherever low-rise buildings are more common. Although good performances pertaining to storm water management can be expected, the sewers should not be downsized (e.g., decreasing diameters), to face possible faults of the RWH systems. In addition, a sensitivity analysis of the influence of the first flush on storm water management could be conducted in future research. Irrigation and street washing require a lower water consumption, thus about 80% of it can be saved. Although not done in this study, it is possible to consider a mix of the different uses: for instance, irrigation and street washing uses could be almost completely satisfied by rainwater.

The economic and practical feasibility of RWH systems are two of the most important problems that limit their implementation in existing settlements [5]. The space required to install a tank can also be a drawback and, especially for private uses, an RWH scheme that groups a small number of buildings (e.g., building blocks) might be more feasible and useful than having one RWH system for each building.

The value of the total storage capacity required for each rainwater use is a rough index of the costs, however the cost quantification of such systems is more complex (e.g., restoration works to build rainwater distribution plants inside buildings, the construction of collection networks to link the buildings involved in RWH for public use, etc.). Moreover, the benefits are not limited to the reduction of the water footprint, the mitigation of damages due to urban flooding and economic savings. A feasibility analysis should also consider Nature-Based Solutions (e.g., SUDS, LID) as real alternatives to classical tanks.

In this paper, some of the approximations that were introduced to resolve the problem of the lack of data may have affected the results: the scarcity of data mainly pertains to the statistical data of the building features and the data on the green areas, thus improvements in national and municipal statistical databases are desirable. In this way, future studies could obtain more robust results to provide a valid tool for municipality planning.
